# 2115. Rezafungin Activity against Invasive Candidiasis Isolates Globally: Results from the 2022 Rezafungin Surveillance Program

**DOI:** 10.1093/ofid/ofad500.1738

**Published:** 2023-11-27

**Authors:** Cecilia G Carvalhaes, Paul Rhomberg, Abby Klauer, Mariana Castanheira

**Affiliations:** JMI Laboratories, North Liberty, IA; JMI Laboratories, North Liberty, IA; JMI Laboratories, North Liberty, IA; JMI Laboratories, North Liberty, IA

## Abstract

**Background:**

Rezafungin (RZF) is a new echinocandin (ECH) approved by the US FDA to treat candidemia and invasive candidiasis (IC). Fluconazole (FLC) resistance (R) is a raising concern to treat IC, and ECHs are often used as first-line therapy. We evaluated the *in vitro* activity of RZF, caspofungin (CSF), micafungin (MCF), anidulafungin (ANF), and azoles against a global collection of 500 *Candida* isolates causing IC.

**Figure:**
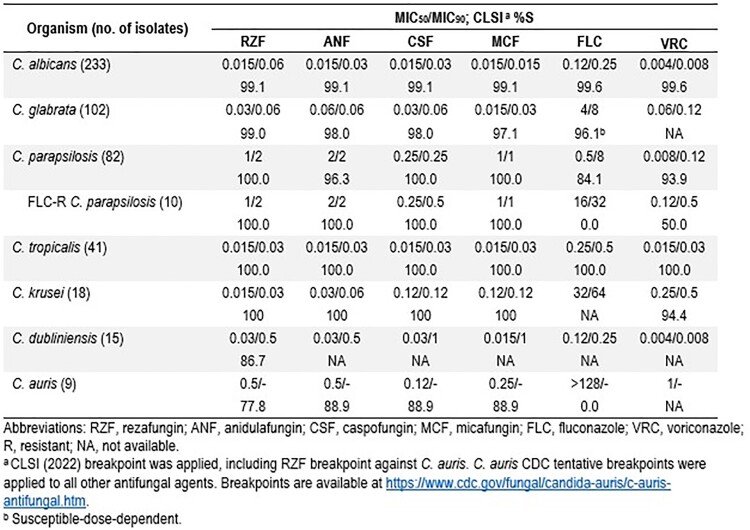

**Methods:**

*C. albicans* (CA; 233 isolates; 46.6%), *C. glabrata* (CG; 102; 20.4%), *C. parapsilosis* (CP; 82; 16.4%), *C. tropicalis* (CT; 41; 8.2%), *C. krusei* (CK; 18; 3.6%), *C. dubliniensis* (CD; 15; 3.0%), and *C. auris* (CARS; 9; 1.8%) isolates were collected (1/patient) in 2022 from 52 medical centers located in Europe (EU; *n*=203; 19 centers), North America (NA; *n*=167; 14 centers), Asia-Pacific (AP; *n*=63; 8 centers), and Latin America (LA, *n*=67; 7 centers), identified by MALDI-TOF MS and/or sequencing and tested by CLSI broth microdilution. CLSI breakpoints (BP) were applied where available. CARS CDC tentative BPs were applied.

**Results:**

RFZ exhibited activity against CA (Table) inhibiting 99.1% of the isolates overall, and 98.6%, 99.0%, 100%, and 100% of isolates from NA, EU, AP, and LA. Only 1 CA isolate (from US) displayed a FLC MIC value within the susceptible-dose dependent (SDD) category. CSP, ANF, and MCF showed S rates of 99.1% against CA. All but 1 CG isolate was susceptible (S) to RZF (99.0%S). CG S rates to CSP, ANF, MCF, and FLC were 98.0%, 98.0%, 97.1%, and 96.1% (SDD), respectively. All CP isolates were S to RZF, CSF, and MCF. However, only 84.1% of CP isolates were S to FLC. RZF and other ECHs inhibited all FLC-R isolates (10 total, 9 EU and 1 US). All CT and CK isolates were S to RZF and other ECHs. All CT were also S to azoles, and 94.4% of CK were S to VRC. Only RZF BPs are available by CLSI against CD (86.7%S). All CARS isolates (9 total; 4 EU, 3 NA, and 2 LA) were FLC-R; but 77.8% and 88.9% were S to RZF and other ECHs, respectively.

**Conclusion:**

RZF demonstrated potent *in vitro* activity against invasive candidiasis isolates regardless of the region, and remained active against FLC-R CP and most of the FLC-R CARS isolates. Based on MIC_50_ and MIC_90_ results, RZF had similar activity to the other ECHs overall.

**Disclosures:**

**Cecilia G. Carvalhaes, MD, PhD**, AbbVie: Grant/Research Support|bioMerieux: Grant/Research Support|Cipla: Grant/Research Support|CorMedix: Grant/Research Support|Melinta: Grant/Research Support|Pfizer: Grant/Research Support **Paul Rhomberg, BS, MT(ASCP)**, bioMerieux: Grant/Research Support|Melinta: Grant/Research Support|Pfizer: Grant/Research Support **Abby Klauer, BS**, Melinta: Grant/Research Support **Mariana Castanheira, PhD**, AbbVie: Grant/Research Support|Basilea: Grant/Research Support|bioMerieux: Grant/Research Support|Cipla: Grant/Research Support|CorMedix: Grant/Research Support|Entasis: Grant/Research Support|Melinta: Grant/Research Support|Paratek: Grant/Research Support|Pfizer: Grant/Research Support|Shionogi: Grant/Research Support

